# Identification of liver metastases with probe‐based confocal laser endomicroscopy at two excitation wavelengths

**DOI:** 10.1002/lsm.22617

**Published:** 2016-12-19

**Authors:** Crispin Schneider, Sean P. Johnson, Kurinchi Gurusamy, Richard J. Cook, Adrien E. Desjardins, David J. Hawkes, Brian R. Davidson, Simon Walker‐Samuel

**Affiliations:** ^1^ Division of Surgery & Interventional ScienceUniversity College LondonFloor 9, Royal Free HospitalLondonNW3 2QGUK; ^2^ UCL Centre for Advanced Biomedical Imaging, Paul O'Gorman BuildingUniversity College London72 Huntley StreetLondonWC1E 6DDUK; ^3^ Department of Tissue Engineering and Biophotonics, King's College LondonDental Institute—Central OfficeFloor 18, Guy's Tower, Guy's HospitalLondonSE1 9RTUK; ^4^ Department of Medical Physics and Biomedical EngineeringUniversity College LondonMalet Place Engineering Building, Gower StreetLondonWC1E 6BTUK; ^5^ Centre for Medical Image ComputingUniversity College LondonThe Front Engineering Building, Floor 3, Malet PlaceLondonWC1E 7JEUK

**Keywords:** liver metastases, confocal laser endomicroscopy, virtual histology, fluorescence microscopy, indocyanine green, fluorescein

## Abstract

**Background:**

Metastasis of colorectal cancer to the liver is the most common indication for hepatic resection in a western population. Incomplete excision of malignancy due to residual microscopic disease normally results in worse patient outcome. Therefore, a method aiding in the real time discrimination of normal and malignant tissue on a microscopic level would be of benefit.

**Material and Methods:**

The ability of fluorescent probe‐based confocal laser endomicroscopy (pCLE) to identify normal and malignant liver tissue was evaluated in an orthotopic murine model of colorectal cancer liver metastasis (CRLM). To maximise information yield, two clinical fluorophores, fluorescein and indocyanine green (ICG) were injected and imaged in a dual wavelength approach (488 and 660 nm, respectively). Visual tissue characteristics on pCLE examination were compared with histological features. Fluorescence intensity in both tissues was statistically analysed to elucidate if this can be used to differentiate between normal and malignant tissue.

**Results:**

Fluorescein (488 nm) enabled good visualisation of normal and CRLM tissue, whereas ICG (660 nm) visualisation was limited to normal liver tissue only. Fluorescence intensity in areas of CRLM was typically 53–100% lower than normal hepatic parenchyma. Using general linear mixed modelling and receiver operating characteristic analysis, high fluorescence intensity was found to be statistically more likely in normal hepatic tissue.

**Conclusion:**

Real time discrimination between normal liver parenchyma and metastatic tissue with pCLE examination of fluorescein and ICG is feasible. Employing two (rather than a single) fluorophores allows a combination of qualitative and quantitative characteristics to be used to distinguish between hepatic parenchyma and CRLM. Lasers Surg. Med. 49:280–292, 2017. © 2016 The Authors. *Lasers in Surgery and Medicine* Published by Wiley Periodicals, Inc.

**Abbreviations**
CI: confidence interval
CLE: confocal laser endomicroscopy
ICG: indocyanine green
pCLE: probe‐based confocal laser endomicroscopy
CRLM: colorectal cancer liver metastasis
ROC: receiver operating characteristics
RFU: relative fluorescence unit
GLIMMIX: generalised linear mixed model analysis by SAS™
SE: standard error

## INTRODUCTION

Colorectal cancer is one of the most common cancers globally, being the third most commonly diagnosed cancer in men and second most common in women 1. Up to a 50% of patients develop liver metastases at some stage 2, a condition which contributes to cancer related mortality in about half of the affected patients 3. Therapies directed at liver metastases have, therefore, been subject of extensive research interest 4.

Surgical resection of solid tumour metastases is the preferred treatment for curative intent 5, 6, resulting in 5‐year survival rates approaching 60% 4. Incomplete tumour resection occur in up to 17% of cases and detrimentally affect patient survival 7, 8. Macroscopically, colorectal cancer liver metastases (CRLM) usually exhibit a white appearance that makes larger nodules easy to distinguish from normal liver tissue, which appears brown.

Unfortunately, visual assessment is insufficient to confirm complete tumour excision. Although intraoperative ultrasonography (IOUS) and bi‐manual palpation are frequently used to determine tumour location within the liver, this approach is not suitable to identify residual cancer following resection because it is mainly of a microscopic nature 9, 10. Identification of residual disease using histopathology is not feasible during liver resection. An imaging modality that can confirm or refute the presence of malignancy within liver tissue at a microscopic level would, therefore, be helpful in ensuring complete resection, reducing the amount of liver tissue resected, or to avoid an unnecessary excision if cancer can be excluded 11.

Other imaging modalities such as cone beam CT, magnetic resonance imaging and near infrared fluoroscopy may be able to enhance imaging during liver surgery, but none of these modalities provides an image resolution that would enable identification of residual disease at a microscopic level 12, 13, 14.

Optical imaging modalities offer micrometer scale image resolution but at this stage only optical coherence tomography (OCT) and confocal laser endomicroscopy (CLE) are available in compact designs that make an intraoperative application feasible. Either imaging modality can visualise liver tissue *ex vivo*, but only CLE has been extensively studied in an *in vivo* setting 15, 16, 17, 18. When employed in a compact design, CLE has a better lateral resolution than OCT at 1.4 versus 7 μm 19, 20. This is an important advantage of CLE which may allow imaging at subcellular level in greater detail. CLE can be performed with a bulkier rigid endomicroscope which contains the required optics in its tip 21 or with a thin, flexible fibre‐based endomicroscope that conducts the excitation laser and transmits signals to an external laser scanning unit 17, 22. For the latter, a commercially available solution is the Cellvizio™ system (Mauna Kea Technology, Paris) which can be used in conjunction with fibre‐optic probes ranging from 0.3–4.5 mm diameter.

Studies comparing confocal laser endomicroscopy (CLE) with standard histology in animal models, have proven its ability to visualise the cellular architecture of tissues, thereby, providing a method of obtaining virtual, *in vivo* histology in real‐time 16, 23. In clinical practice, probe‐based confocal laser endomicroscopy (pCLE) is licensed for the endoscopic diagnosis of gastrointestinal, bronchial and urinary malignancy and dysplasia. Depending on the tissue type, diagnostic accuracy is in the range of 86–91% 24, 25, 26, 27. Recent reports suggest that pCLE may also be a useful tool for the detection of breast and head and neck cancers 28, 29.

To the best of our knowledge, the detection of liver metastasis using pCLE has not been investigated previously. Therefore, this study was conducted to assess to what extent dual wavelength pCLE imaging characteristics and quantitative fluorescence properties could discriminate between metastatic and normal liver tissue in an orthotopic nude mouse model of colorectal cancer liver metastases. Dual wavelength CLE imaging was achieved by recording measurements with the Cellvizio™ 488 and 660 nm system employing either fluorescein or indocyanine green as fluorophores, respectively. Both fluorophores are clinically licensed which enables findings to be translated into a clinical context without safety concerns.

## METHODS

### Animal Model

All animal studies were approved by the University College London Biological Services Ethical Review Committee and licensed under the UK Home Office regulations and the Guidance for the Operation of Animals (Scientific Procedures) Act 1986 (Home Office, London, United Kingdom) and United Kingdom Co‐ordinating Committee on Cancer Research Guidelines for the Welfare and Use of Animals in Cancer Research 30. The human colorectal carcinoma cell line SW1222 was cultured under aseptic technique in Dulbecco's Modified Eagles Medium containing 5 mM l‐glutamine and 10% v/v foetal bovine serum. All animal studies were conducted in accordance with UK home office regulations and the guidelines for the welfare and use of animals in cancer research. The liver metastasis model used has been previously described 31. Briefly, eight (*n* = 8) MF1 *nu/nu* mice (female, 6–8 weeks old, 25–30 g) underwent a laparotomy in order to directly inject 1 × 10^6^ cells into the spleen. Splenectomy was performed after 5 minutes to stop formation of a primary tumour at the site of injection, with cells that had been carried by the blood flow through to the liver forming the solid tumours of the metastatic model. At conclusion of the procedure, the laparotomy incision was closed and animals were recovered. In the described animal model, female mice are exclusively used due to their better compliance.

Liver tumour maturation took between 5 and 6 weeks. The progression of metastatic disease was monitored once per week with non‐invasive magnetic resonance imaging under general anaesthesia. The evolving disease pattern showed a heterogeneous distribution of distinct solid tumour nodules throughout the lobes of the liver, typically comprising 10–20 tumour nodules that could be visualised on magnetic resonance imaging.

### CLE Image Acquisition

A transverse laparotomy was carried out under general anaesthesia to allow unrestricted access to the liver. CLE imaging at 488 and 660 nm was performed with two different CLE imaging stacks with separate laser scanning units, probes and image processing computers. Both systems were initialised and calibrated at the start of each experiment. Switching between wavelengths simply required imaging probes to be swapped over. Due to the inability of pCLE to visualise tissues beyond 100 μm depth, images were exclusively acquired from superficial tissue, while the probes were manually maintained in contact with the liver surface. Motion artefact was reduced by gently applying the pCLE probes onto the liver surface, so that they moved in parallel with liver motion secondary to respiration.

Images were acquired with the Z‐probe at 488 nm and the mini‐Z probe at 660 nm wavelength. Probe characteristics were the following: probe diameter 1.8 or 0.94 mm; lateral resolution 3.9 μm, axial resolution 44 or 13 μm, working distance 80 or 65 μm, maximal field of view 426 × 302 or 323 × 323μm for the Z‐probe or mini‐Z probe, respectively.

The liver was macroscopically categorised into normal and cancerous tissue areas based on its respective brown or white appearance. This approach was made possible by the radically different appearance of normal liver versus CRLM. Image sequences that were disturbed by excessive motion artefact were not used, especially since they would frequently included images from normal and cancerous tissues at the same time. No attempt was made to identify microscopic disease because the aim was to establish reproducible CLE criteria of CRLM which could then be translated to the detection of microscopic disease in future studies. Baseline CLE images were recorded over a representative area prior to fluorophore administration. To provide exogenous fluorescence for CLE imaging at 488 and 660 nm excitation, fluorescein sodium (molecular weight 376.27 g/mol—henceforth called “fluorescein”) and ICG (molecular weight 774.96 g/mol) were mixed with 0.9% sodium chloride or 5% dextrose, respectively. Subsequently, fluorescein (4–7 mg/kg) and indocyanine green (0.3–0.5 mg/kg) were sequentially injected into the tail vein with a delay of 20–40 minutes between injections to allow adequate time for imaging. In preliminary experiments, it was confirmed that neither fluorescein nor ICG elicited unintentional “cross”‐fluorescence signals.

An initial image sequence of 1–3 minutes was recorded to monitor and confirm distribution of the fluorophores in the liver. Several short sequences ranging from 10–20s were taken from normal and malignant tissue in different locations. The aim was to acquire images from as many tumour nodules, and an equivalent number of normal liver tissue areas, as possible. It was not feasible to co‐register tissue areas between 488 and 660 nm pCLE imaging. But due to the relatively small size of mouse livers, it was hypothesised that approximately, the same areas of view were imaged at both wavelengths. For statistical analysis which is described below, all values for individual animals were pooled into one group and hence co‐registration is less relevant than for the visual comparison of image features. Pooling of data was also intended to decrease the impact of heterogeneous fluorophore accumulation and tissue scattering properties within the same animal. The mean fluorescence values for each image frame from these sequences were subsequently analysed.

### Preparation and Examination of Tissue Samples

Following termination of the animals, the livers were resected, fixed in 10% formalin and embedded in paraffin blocks. Tissue samples were sliced at 4 μm thickness with a microtome and sections mounted on glass slides. The presence of colorectal cancer metastases was confirmed by using a standard structural Haematoxylin and Eosin stain. Because areas of normal liver and CRLM in this disease model looked radically different, histological examination enabled us to confirm infiltration of adenocarcinoma cells in representative liver areas which previously underwent CLE *in vivo* examination.

### Statistical Analysis

Cellvizio™ is packaged with image analysis software (Imagecell™) that records and displays basic values termed relative fluorescence units (RFU), from which the maximum, minimum, median and mean were calculated and exported for each image frame. Relative fluorescence units are based on a reconstruction of single fibre fluorescence intensity at a given point in time. A number of factors such as fibre injection rate, fibre collection rate, fibre auto‐fluorescence, biological sample fluorescence and laser source intensity influence fluorescence intensity. These variables are factors in an equation that allows the real time reconstruction of fluorescence intensity in individual fibres each of which corresponds to an image pixel of the CLE image. A detailed description of the equation and other relevant methodology has been previously published 32. At the start of each imaging session, a calibration is carried out using solution that provides a uniform value of background fluorescence. The calibration procedure results in homogenisation of the individual probe fibres to give them uniform detection sensitivity (which corresponds to a measured light intensity) 33. This process enables comparison of fluorescence values between individual fibres (i.e. pixels) of the probe, for a given calibration. However, whilst the absolute background fluorescence value in the calibration solution is uniform it may change between repeated calibrations and different solution containers, meaning that fluorescence units have to be regarded as relative, as opposed to absolute values. Thus, imaging data were only compared within the same animal.

Negative RFU values either correspond to image noise or can be caused by a reduction in fibre autofluorescence due to prolonged illumination. To avoid misinterpretation, negative RFU values were thresholded at zero. All RFU values underwent a square root transformation to reduce data skew and kurtosis as previously reported 20.

Routine statistical comparison, e.g. by paired Wilcoxon rank sum test (individual animal paired with RFU) was not possible due to specific constraints of the acquired data which were: (1) unequal number of frame counts for normal and CRLM tissue and (2) potential introduction of a repeat sampling error. These circumstances lead us to choose a generalised linear mixed model (GLIMMIX) analysis which can accommodate for the described data constraints by estimating the interaction of fixed and random effects. The GLIMMIX analysis enabled modelling of the probability of the acquired images corresponding to normal liver parenchyma. The measured RFU value and animal ID were set up as fixed effect and random effect for this model, respectively. A similar approach has previously been used by our group for CLE evaluation of ablated liver tissue in a porcine model 20.

Different estimation methods such as Pseudo Likelihood, Laplace and Gauss‐Hermite quadrature can be used for GLIMMIX analysis. For the purpose of this study, the Laplace method was chosen because it exhibited satisfactory convergence and the best fit characteristics as assessed by Akaike information criterion (AIC). GLIMMIX analysis results are given as the change in odds ratio for an increase in RFU at a standardised value. For example, an odds ratio of 2 at a standardised RFU increase of 100–101 means that the odds ratio of liver parenchyma being normal increases by 2 if the RFU value increased by one from 100 to 101.

To test the validity of the GLIMMIX model resulting from the study data, a ROC analysis was performed. The covariates and intercepts provided by the GLIMMIX analysis were used to calculate the log odds, odds and finally, the probability of identifying normal liver tissue. Each single probability value corresponded to a single observation (mean RFU per frame), a specific animal ID and a tissue type which enabled us to perform a ROC analysis based on this data. Because animal ID was a random effect due to the difference in probe calibrations between experiments, the same RFU value would result in different probability values for individual animals.

To determine if a probability based threshold value could be used to distinguish between normal and malignant tissue random sample cross validation was carried out. To this end, all observations were randomised into a training and a validation dataset. The training set was used to build a GLIMMIX prediction model whose output subsequently underwent ROC analysis. A threshold value with an optimal sensitivity and specificity for identifying normal liver tissue was then chosen from the ROC curve coordinates. This threshold probability value was subsequently applied to the validation set, to evaluate its performance in distinguishing between normal and malignant liver tissue and calculate the resulting sensitivity, specificity and accuracy for this test.

An estimation of erythrocyte flow velocity calculated from the available image data was also carried out. To calculate flow velocity (in mm/s), the distance travelled by a single erythrocytes in the time period between two image frames (=41.7 ms at 24 frames per second) was measured. Depending on the amount of available data, 5–10 individual erythrocytes were assessed per tissue type and animal ID.

Statistically significant differences were assumed if *P* < 0.05. Statistical analysis was performed using SPSS™ Version 21 (IBM, Armonk, NY) with exception of the GLIMMIX analysis which was carried out with SAS™ Version 9.4 (SAS Institute, Cary, NC). Quantitative data (RFU and erythrocyte flow velocity) is given as median and interquartile range. Odds ratios are stated together with their 95% confidence intervals (CI).

## RESULTS

### Test Sample Size

The orthotopic CRLM model was established in eight mice. One animal died before any CLE measurements could be carried out. The remaining seven mice underwent CLE examination. In two animals, the tumour burden relative to normal liver was too great to allow meaningful statistical comparison. Two animals died during image acquisition; one (ID6) before sufficient data at either wavelength could be collected and a further animal following completion of CLE imaging with fluorescein at 488 nm (ID7). In summary, six animals underwent consequential CLE imaging and of these, four (fluorescein/488 nm) and three (ICG/660 nm) experiments provided sufficient data for statistical analysis.

### Evaluating Normal Liver at 488 nm Wavelength With Fluorescein

Examples of CLE images and H&E histology for normal and CRLM tissue are shown below. Each example is from the same animal but due to the frequently small size of lesions, no attempt was made to spatially correlate CLE images with histological slides. Macroscopic lesion size was larger than the pCLE field of view but otherwise variable between animals. The smallest lesions were 1–2 mm whereas in some animals, the majority of the liver surface was covered by metastatic deposits.

**Figure 1:**
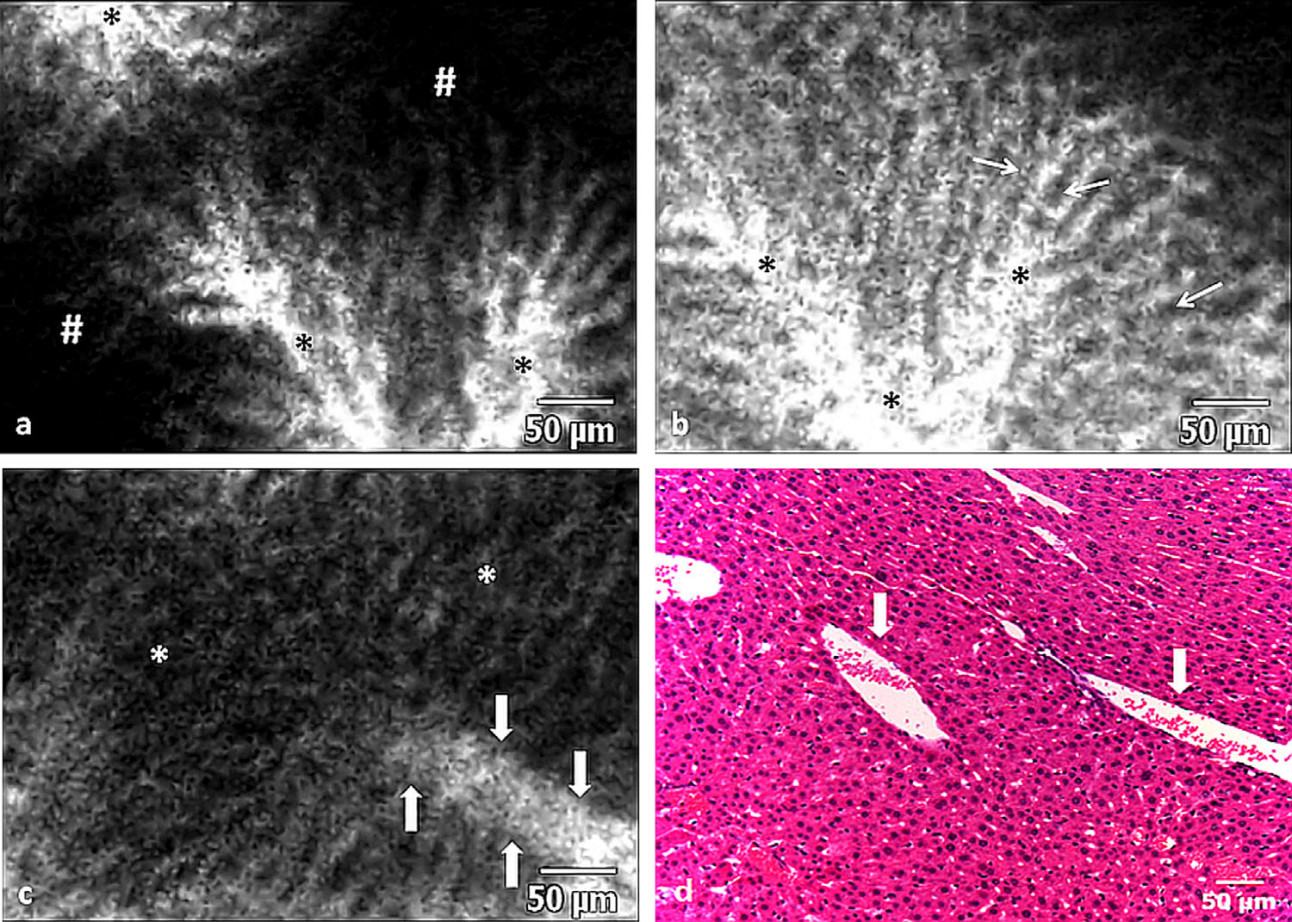
(**a**) A CLE image (488nm) showing fluorescein entering the intravascular space (*) shortly after injection. (**b**) At completion of the inflow phase (>12 minutes after fluorophore injection), hepatocyte cords appear dark (arrows), whereas the intravascular space which includes sinusoids and larger lobular vessels is bright (*). (**c**) Erythrocytes can be seen as dark shapes within the bright intravascular space (arrows outline the blood vessel containing the erythrocytes). The remainder of the image shows the typical pattern of normal liver parenchym consisting of a mixture of hepatocyte cords and sinusoids. (**d**) Histology of normal liver tissue with two blood vessels (arrows) containing erythrocytes.

Prior to administration of fluorescein, no endogenous fluorescence was observed in normal or metastatic tissue. The initial period following fluorescein injection was termed the inflow phase. During this phase, the fluorescein strongly accumulated within the vascular compartment and could be observed in hepatic sinusoids and interlobular vessels (Fig. 1a). Occasionally, individual erythrocytes could be visualised moving through blood vessels or sinusoids. Because erythrocytes did not take up fluorescein, they could be seen as small, dark, round or discoid shapes outlined against the bright contrast signal of the intravascular fluorophore (Fig. 1b). Generally, inflow phase images were dominated by a meshwork pattern of bright interlobular vessels criss‐crossing dark areas which represented the parenchyma of liver lobules (Fig. 1c).

**Figure 2:**
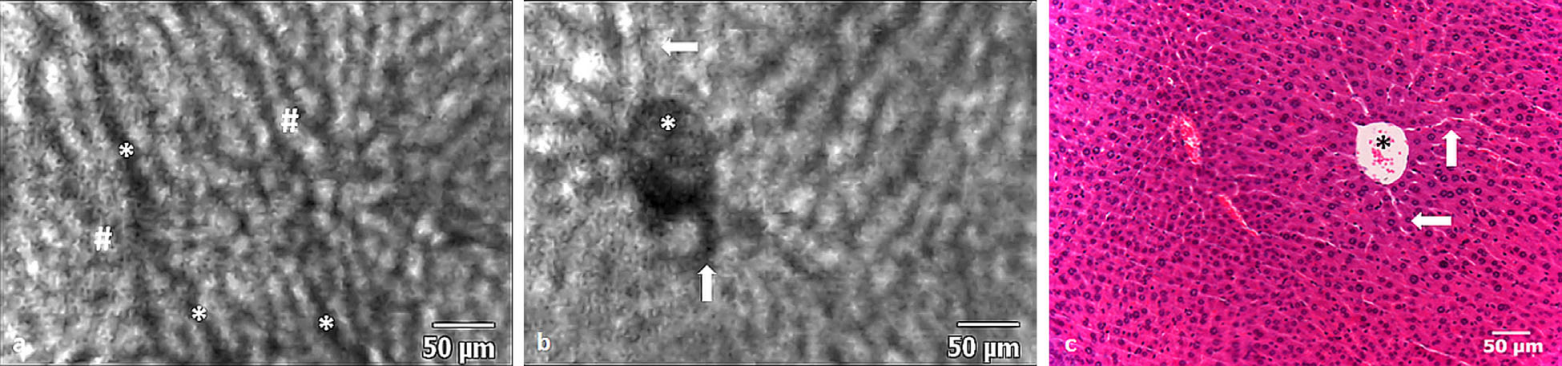
(**a**) In the parenchymal phase of fluorescein administration, hepatocytes appear bright (#marking examples) in CLE images (488 nm) whereas the intravascular space (*) is dark due to extravasation of the fluorophore into the interstitium. (**b** and **c**) The central vein (*) can be visualised as a round area in the middle of the hepatic lobule with feeding sinusoid vessels (arrows) converging towards it. (**c**) Histology with a similar configuration to the CLE image in b.

Inflow phase duration varied between animals but was approximately 7–11 minutes. At the end of the inflow phase, the fluorophore shifted from the intravascular compartment to the liver parenchyma and connective tissue, which resulted in high signal intensity within liver lobules. This stage of the CLE examination, lasting throughout the remainder of observations, was termed the parenchymal phase. Throughout its duration, cords of hepatocytes and the sinusoid structure could frequently be observed (Fig. 2a). Sinusoids and other liver vessels appeared largely dark and devoid of fluorescence, and this phenomenon became more marked at the later stages of image acquisition.

Differentiation between types of vasculature (e.g. portal vs. central venous vessels) was challenging at either imaging phase but could sometimes be accomplished by interpreting the vessel's location and morphology. For example, a central vein (which drains towards hepatic vein) could be identified by its location and orientation within the middle of a liver lobule (Fig. 2b).

### Evaluating Liver Metastases at 488 nm Wavelength With Fluorescein

**Figure 3:**
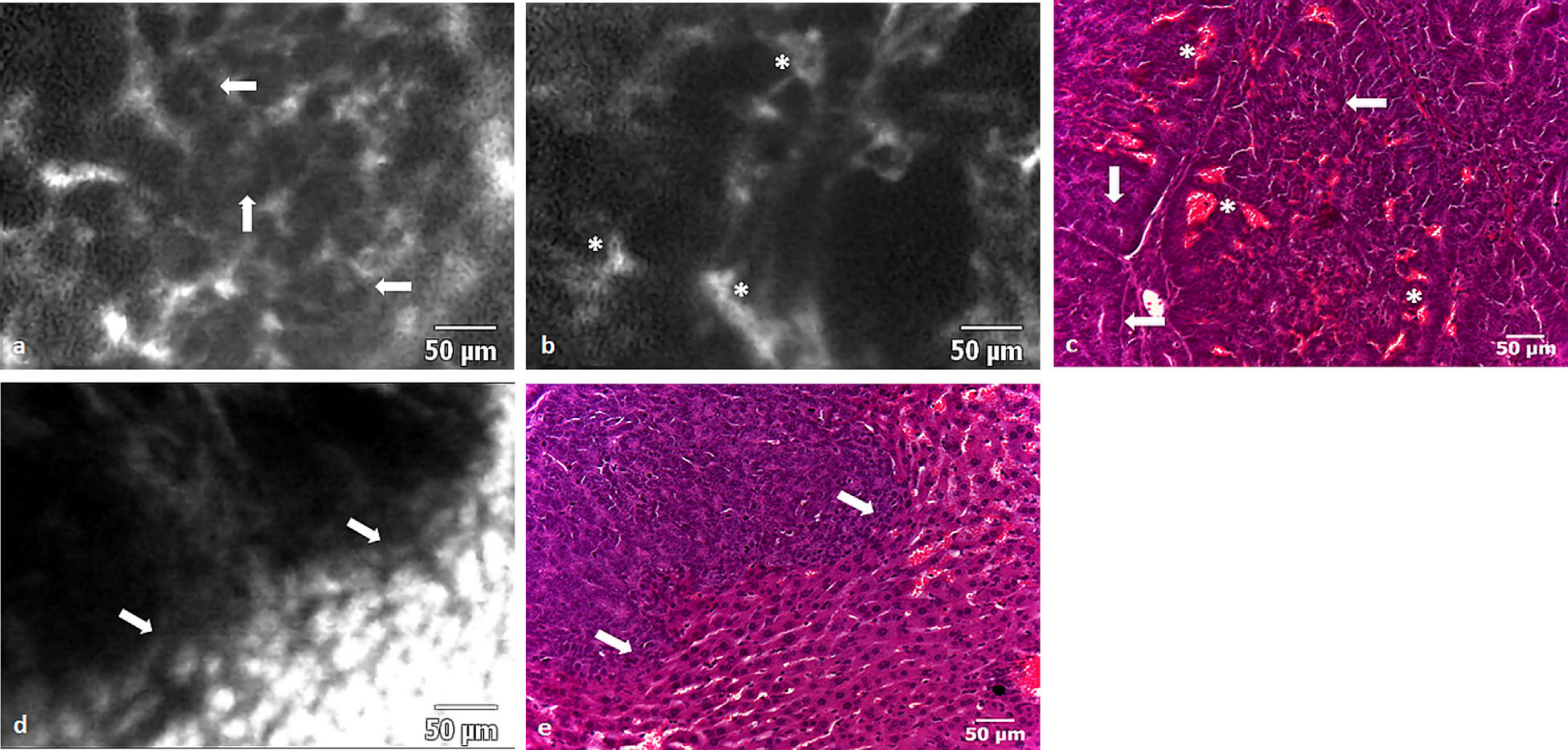
(**a**) In the mouse model, fluorescein enhancement in CRLM tissue during the parenchymal phase was characterised in CLE images by dark, irregular, round structures that retain some of the original glandular architectural features that can also be seen on histology. The concentric arrangement of adenocarcinoma cells (arrows) results in the “bullseye” appearance of a dark ring around a bright centre on CLE and H&E histology. (**b**) Irregularly arranged vessels containing erythrocytes (*) can be seen throughout the tumour tissue. In contrast to normal liver tissue, the intravascular space within CRLM retained the circulating fluorophore throughout the entirety of the observation period. (**c**) The corresponding histology features showing concentrically arranged adenocarcinoma cells (arrows) and irregular vessels (*). (**d**) The border (arrows) between normal (bright) and cancerous tissue (dark) can be readily appreciated on CLE imaging. (**e**) Border between normal and cancerous tissue on H&E histology.

Areas of CRLM could be analysed in detail at 488 nm wavelength using fluorescein. The majority of malignant tissue showed a pattern of dark patches criss‐crossed by bright linear structures. The areas devoid of fluorescence represented malignant tissue which was interspersed by torturous, neoangiogenic vessels that traversed throughout the metastasis. Fluorescein appeared to be retained within tumour vessels for longer than in the healthy hepatic vasculature. This resulted in neoplastic vessels displaying high intensity fluorescence throughout most of the image acquisition. Infrequently, glandular structures typical of moderate to well differentiated colonic malignancy could be visualised (Fig. 3a).

Visible erythrocytes exhibited a mostly slow and erratic movement when compared to normal hepatic vessels (Fig. 3b and c). In two animals, there was sufficient image data to estimate erythrocyte flow velocity in both tissue types. Median flow velocity in normal tissue was 0.47 ± 0.13 and 0.52 ± 0.31 mm/s compared to 0.18 ± 0.15 and 0.15 ± 0.1 mm/s in CRLM tissue for animal ID1 and ID8, respectively. Subjective discrimination between normal and malignant tissue was feasible and a demarcation line could be visualised in all animals (Fig. 3d and e).

### Evaluating Normal Liver at 660 nm Wavelength With ICG

**Figure 4:**
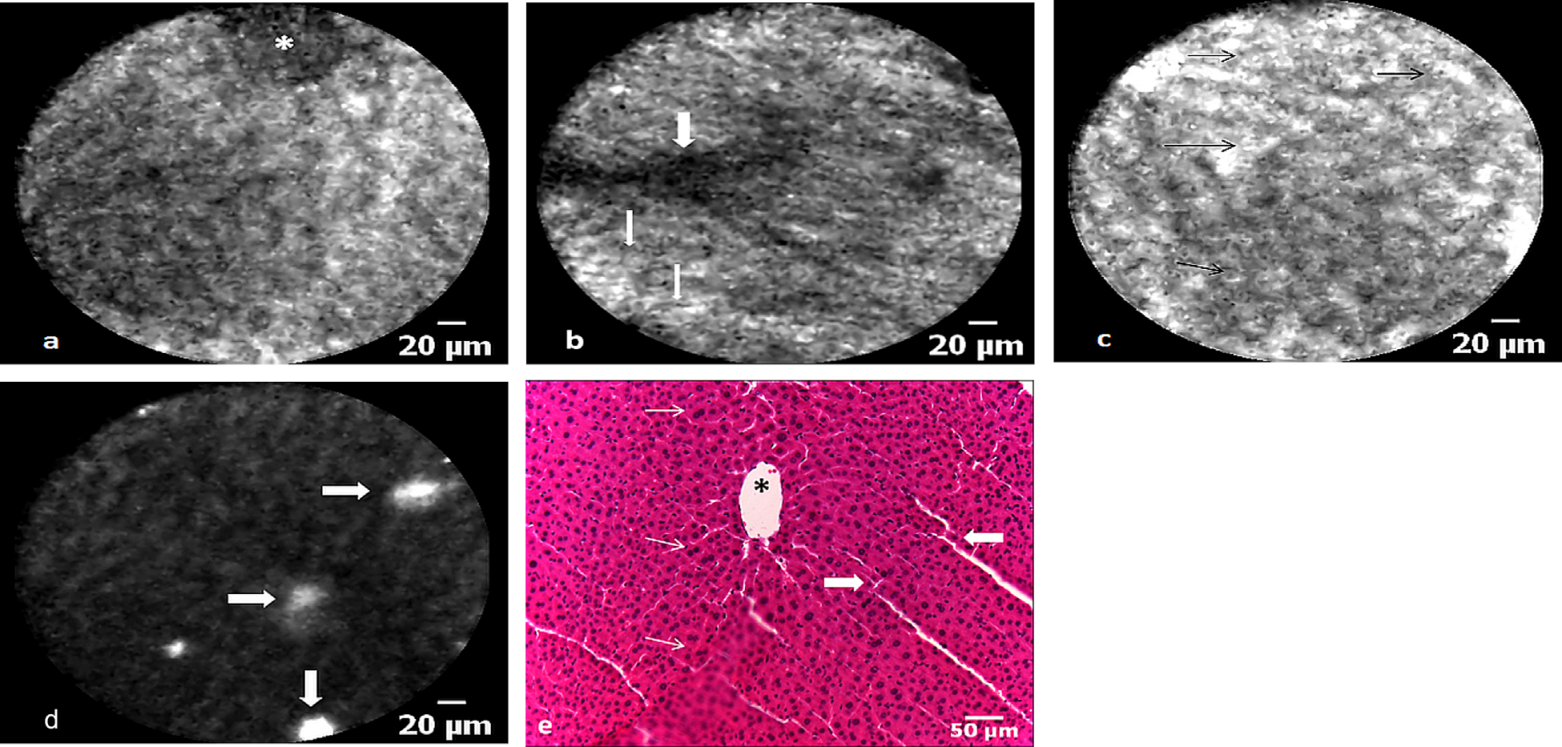
(**a**) A central vein (*), acquired *in vivo* with CLE (660 nm), surrounded by normal parenchyma, acquired following ICG administration, which can also be seen in a corresponding H&E image. (**b**) The distribution pattern of ICG mediated fluorescence enables discrimination between sinusoids (thin arrows), larger blood vessels (wide arrow), and the hepatocytes, the latter of which make up most of the image. Vessels and their bifurcations are represented by dark linear structures (arrows). (**c**) Hepatocyte nuclei can be seen as small contrast sparing areas (thin arrows), surrounded by bright cytoplasm. (**d**) At the end of the parenchymal phase, hepatocytes have lost most of their fluorescence. Bright regions (arrows) spaced throughout the normal tissue likely presents areas where bile juice is concentrated (e.g. bile ducts). (**e**) Section of normal liver histology depicting a central vein (*), nuclei (thin arrows), and larger interlobular vessels (wide arrows).

CLE examination at 660 nm did not reveal any endogenous fluorescence in areas of normal liver or CRLM tissue prior to ICG injection. In contrast to CLE imaging at 488 nm with fluorescein, no signal was detectable from blood vessels during the inflow phase following ICG administration, and erythrocyte movement could not be visualised. In the parenchymal phase, however, structures with the configuration of central lobular veins could be identified (Fig. 4a). These had the typical appearance of round, irregularly outlined structures, situated in the centre of the lobule. During the parenchymal phase, ICG's high specificity for the cytoplasm of hepatocytes, resulted in good visualisation of sinusoidal structures and allowed distinction between hepatocytes and the surrounding vasculature (Fig. 4b).

In general, blood vessels were identified as linear or round structures that had a lower fluorescence signal, relative to the surrounding hepatocytes. Because of the prominent cytoplasmatic fluorescence, the nuclei of individual hepatocytes were identifiable as dark intracellular areas (Fig. 4c). Approximately, 15 minutes after injection of ICG, bright regions of around 5–20 μm diameter could be observed throughout the parenchyma. This phenomenon possibly represents areas of ICG accumulation (e.g. bile juice) (Fig. 4d).

### Evaluating Liver Metastases at 660 nm Wavelength With ICG

**Figure 5:**
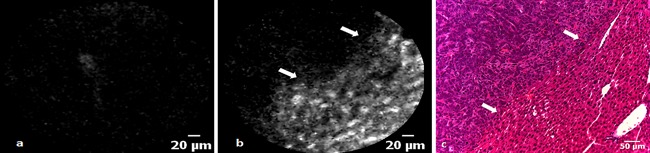
(**a**) A CLE image of a region of CRLM, which is devoid of any fluorescence following ICG administration. (**b**) An irregular transition (arrows) from low to high fluorescence represents the demarcation line between CRLM and normal tissue in a CLE image acquired at 660 nm. (**c**) The border between metastasis and normal liver tissue viewed on H&E histology.

In contrast to CLE imaging of fluorescein at 488 nm, areas of CRLM, as identified by visual inspection, did not emit any measurable fluorescence and, therefore, appeared as dark areas covering several fields of view (Fig. 5a). Although no specific tumour characteristics could be appreciated, it was possible to visualise the delineation between CRLM and normal hepatic tissue by observing a demarcation between high and low intensity fluorescence areas (Fig. 5b).

### Statistical Analysis of Fluorescence Values

**Figure 6:**
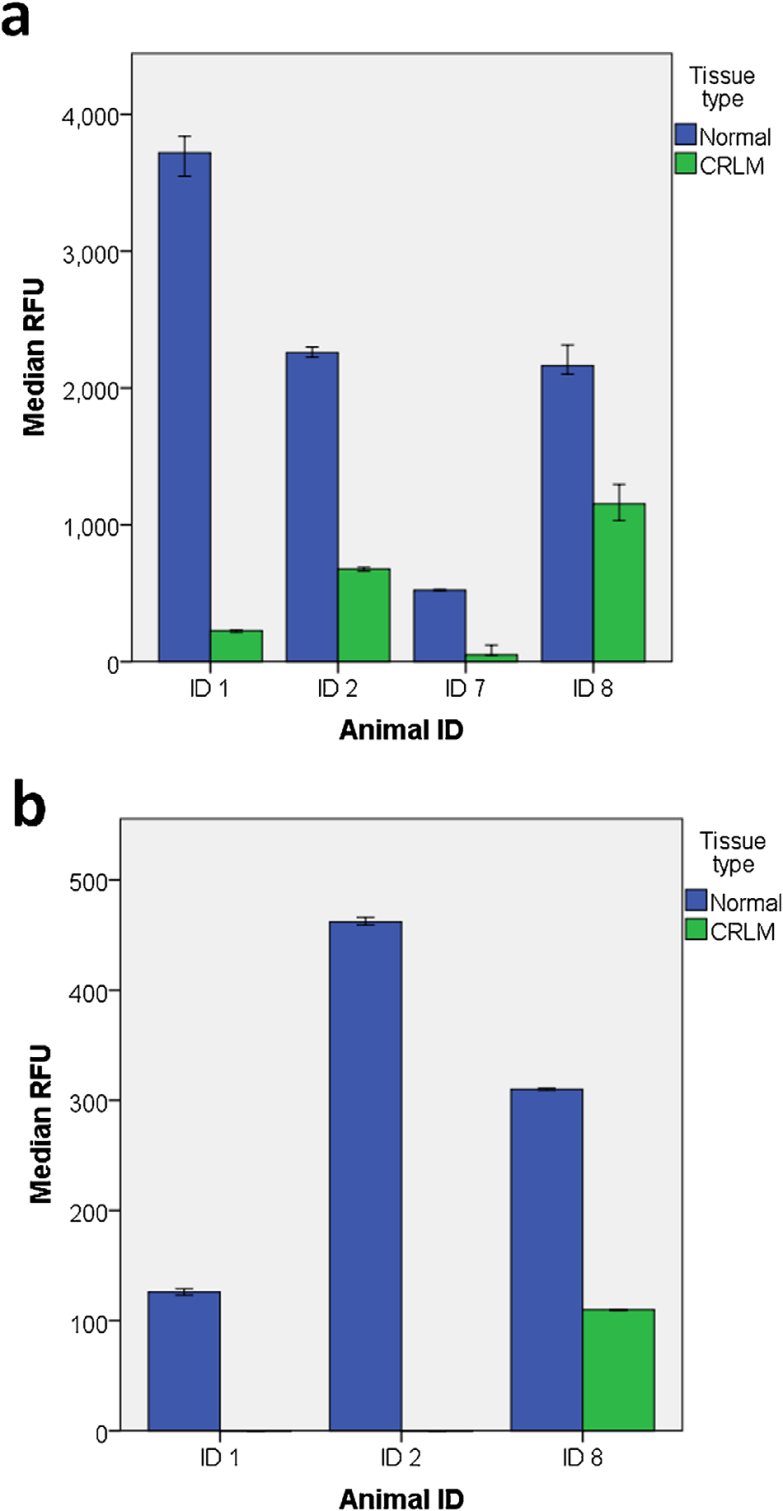
(**a**) Median RFU values ± 95%CI comparison between normal and CRLM tissue for each animal liver imaged at 488 nm with fluorescein. (**b**) Median RFU values ± 95%CI comparison between normal and CRLM tissue for each animal liver imaged at 660 nm with ICG. Note that the median and 95%CI for CRLM tissue in animal ID 1 and ID2 is zero and is, therefore, not displaying on the bar chart.

RFU values measured during the parenchymal phase in metastatic tissue were 53–94% lower for fluorescein/488 nm and 65–100% lower for ICG/660 nm when compared to normal liver tissue (Table 1, Fig. 6). GLIMMIX analysis showed that higher RFU values measured during the parenchymal phase were statistically significant predictors for the presence of normal liver tissue at either fluorophore and wavelength combination.

**Table 1:** Comparison of Relative Fluorescence Units in Normal Liver and Colorectal Cancer Liver Metastasis

	488 nm with fluorescein			660 nm with ICG		
Animal ID	Normal (*n* = 2195)	CRLM (*n* = 1922)	RFU change (%)	Normal (*n* = 2681)	CRLM (*n* = 2050)	RFU change (%)
1	3720 (2857)	226 (216)	94	126 (53)	0 (0)	100
2	2259 (760)	678 (308)	70	462 (150)	0 (0)	100
7	523 (164)	51 (128)	90	*	*	*
8	2164 (1948)	1153 (1763)	53	310 (43)	110 (113)	65

Median RFU values in normal and CRLM tissue during the parenchymal phase are shown for each animal with the interquartile range stated in brackets. Decrease of RFU from normal to CRLM tissue is shown in percent. Negative RFU values were thresholded to zero. *, no data collection as animal expired during experiment.

For the fluorescein/488 nm combination, a RFU value change from 37.8 to 38.8 increased the odds ratio of liver tissue being normal (e.g. nonmetastatic) by 1.2 (*P* < 0.0001, CI 1.21–1.24). This was also true for the ICG/660 nm combination where a RFU value change from 10.5 to 11.5 resulted in an odds ratio increase of 4.6 (*P* < 0.0001, CI 3.8–5.7). The impact of treating individual animals as a random effect did significantly contribute to the fit of either model with a covariance parameter of 3.9 ± 2.8 (SE, *P* < 0.001) for fluorescein/488 nm and 43.0 ± 35.8 (SE, *P* < 0.05) for ICG/660 nm. The covariance parameter estimation indicates to what degree the random variable affects the fit of the mathematical model (e.g. aids in predicting outcome) and its associated standard error gives a measure of repeat sampling variability.

Subsequently, observations were randomised into a training and validation set for each fluorophore and wavelength combination. GLIMMIX analysis of the training samples retained statistical significance with a RFU increase from 37.7 to 38.7 and 10.6 to 11.6 resulting in an odds ratio increase of 1.22 (*P* < 0.0001, CI 1.20–1.24) and 5.08 (*P* < 0.0001, CI 3.62–7.12) for the fluorescein/488 nm and ICG/660 nm combinations, respectively. Again the random effect from performing measurements in different animals did contribute to the fit of the model with a covariance parameter of 3.8 ± 2.7 (SE, *P* < 0.001) for fluorescein/488 nm and 49.2 ± 42.0 (SE, *P *> 005) for ICG/660 nm.

ROC analysis of the training set resulted in an area under the curve of 0.934 ± 0.003 (CI 0.928–0.941) for fluorescein/488 nm and 0.994 ± 0.001 (CI 0.992–0.997) for ICG/660 nm. A probability threshold value of 0.67 for fluorescein/488 nm and 0.69 for ICG/660 nm (representing a fixed RFU value and random animal effect) with a high corresponding sensitivity and specificity was chosen from the ROC curve coordinates. Based on these threshold values, the validation set observations were categorised into either normal or metastatic tissue which consequently enabled calculation of sensitivity and specificity for the proposed quantification method. For the fluorescein/488 nm combination, sensitivity and specificity was 82.9% (CI 81.1–84.6%) and 85.2% (CI 83.2–87.0%) respectively. For CLE imaging of ICG at 660 nm wavelength, the sensitivity and specificity was 97.9% (CI 97.0–98.6%) and 97.5% (CI 96.4–98.4%), respectively.

## DISCUSSION

This article represents the first *in vivo* comparison of dual‐wavelength CLE imaging in combination with two exogenous fluorescent probes as a method of differentiating liver metastases from normal liver parenchyma. The CLE system used in this study allows high‐resolution (3.9 µm), imaging of fluorescence with a field of view of 424 × 302 µm (488 nm) or 323 × 323 µm (660 nm), thereby, allowing *in vivo* tissue structure and function evaluation in real time.

Two distinct phases of CLE liver imaging, based on fluorophore behaviour, have been described. The inflow phase lasted up to 11 minutes and was characterised by accumulation of fluorophore (and hence signal intensity) in the intravascular space. Following on seamlessly, and lasting for the remainder of the CLE examination, the parenchymal phase showed a signal intensity shift to the liver lobules. Images obtained in healthy liver tissue at 488 nm wavelength with fluorescein provided good detail of the sinusoidal structure and hepatic vascular architecture. In contrast, CRLM tissue showed a pattern of tumour cell agglomerations which were traversed by haphazardly arranged blood vessels.

Quantitative estimation revealed that erythrocyte flow velocity in CRLM tissue was slower than in normal liver tissue. In contrast to healthy vessels that relatively quickly lost fluorescence during the inflow phase, the signal intensity in tumour vasculature remained high throughout the inflow and parenchymal phases of imaging. This was intriguing because, commonly, tumour vessels are reported as having increased permeability 34 which can lead to a rapid extravasation from the intra to the extravascular compartment. This might have resulted in rapid transfer of fluorescence from the vasculature to the surrounding cancer tissue, whereas, in fact, the visualised cancer tissue was largely devoid of signal which remains unexplained by our experiments. This finding could potentially be explained by a number of tumour specific or general factors such as local tissue necrosis, systemic hypoperfusion, arterio‐venous shunts, or the unique nature of the tumour cell line. Alternatively, tumour vasculature associated factors such as reduced permeability due to accelerated vessel maturation or volume expansion of the extravascular space causing dilution of fluorophore concentration could also be responsible for the observed lack of fluorescence intensity. Without further studies, it is difficult to put these findings into context because to the best of our knowledge, pCLE imaging of a comparable small animal model has not been reported in the past.

Imaging at 660 nm wavelength with ICG was dominated by the rapid uptake of ICG into the cytoplasm of normal hepatocytes. Visualising the whole field of view enabled sinusoid architecture to be identified, whereas on an intracellular level, hepatocyte nuclei could be observed as dark areas contrasted by the strong cytoplasmatic fluorescence signal. Localised regions of high intensity fluorescence that were seen at the later stage of imaging could not reliably be attributed to a specific feature of liver histology. Because ICG is known to be exclusively excreted by bile 35, it is possible that these regions were small bile ductules where the fluorophore accumulated before being transported to the larger bile ducts. During image acquisition, no vascular contrast could be observed but occasionally vessels could be identified as dark linear or circular structures outlined against the bright surrounding liver parenchyma.

Areas of CRLM were characterised by lack of ICG mediated fluorescence purportedly due to the lack of ICG uptake in the cancerous tissue. The absence of any relevant imaging signal prevented the characterisation of CRLM tissue with this wavelength and fluorophore combination. The general finding was that fluorescein in combination with CLE at 488 nm was more suited to the imaging of the vasculature and for examining CRLM morphology, which was characterised by a mosaic of dark areas, irregular vasculature and, occasionally, glandular structures. In contrast, CLE imaging of ICG at 660 nm appeared to be better suited to assess the structure of sinusoids and the surrounding hepatocytes.

Although some reports on *in vivo* CLE examination of human liver disease have been published to date 17, 36, no data exists describing the application of CLE in liver surgery for cancer. Several of the imaging features described in this animal model, indicate that they could be usefully translated to patients. For example, the combination of glandular architecture and proliferation of irregular vasculature with abnormal flow characteristics, as observed on CLE examination of fluorescein at 488 nm, is a common histological feature of human CRLM 37, 38, 39, 40. A lack of ICG related fluorescence that was apparent at 660 nm has previously been reported using camera based near‐infrared imaging during liver resection for colorectal metastases 14.

The feasibility of using CLE intraoperatively to confirm complete removal of cancer in the resection of intracranial malignancy has been demonstrated previously 41. This approach could also be of benefit during resection of CRLM, where confirmation of malignancy at the borders of a resection may allow a more radical procedure to be performed with the intention of increasing cure rates. If presence of microscopic malignancy can be excluded on the other hand, it would allow surgeons to minimise the resected total liver volume which in turn can reduce the incidence of postoperative complications 42.

The same rationale can also be applied to ablation therapy of CRLM which is emerging as a feasible alternative or auxiliary treatment modality beside surgical resection. In liver ablation, cancerous tissue is destroyed by inducing coagulation necrosis through a variety of methods (e.g. radiofrequency, microwave ablation) 43. Our group has previously demonstrated in a porcine model that laparoscopic pCLE enables discrimination between normal and ablated liver tissue 20. Due to the difficulties of establishing malignancy in a large animal model 44, no pCLE examination of liver cancer was carried out. Because Cellvizio™ and other non‐commercial pCLE systems are not yet licensed for clinical use during surgery, it was crucial to investigate its potential benefit in an animal model before considering its clinical evaluation.

In the current study, it was demonstrated that utilising a dual wavelength approach may be more advantageous than using single wavelength CLE 17, 36, because two fluorophores and their respective imaging properties in tissue can be evaluated in short succession. Fluorescein and ICG CLE visualisation in hepatic tissue differs because the former has properties suited to the visualisation of vasculature 36 whereas the latter is exclusively cleared by the liver and, therefore, has a stronger affinity to hepatocytes and bile ducts 35.

Other groups have explored the potential of CLE imaging for identifying cancers and evaluated these in animal models 16, 45, 46. These, however, have been based on nonclinically approved CLE systems which would need further development before they can be used in a clinical context 23, 45. The experiments outlined here were all performed with a CE marked device that has found widespread interest for the clinical diagnosis of malignancy 26, 47, 48, 49 and it is hoped that the relatively widespread dissemination of this CLE imaging platform can help in facilitating the further clinical evaluation of the findings presented here.

Previous articles on CLE imaging of malignancy have been based on subjective, observer dependent image interpretation but have not identified quantifiable and reproducible parameters of malignancy 23, 50. To establish such a quantifiable parameter that would allow discrimination between normal and malignant liver, a tissue evaluation centred on numerical fluorescence values was proposed. A GLIMMIX analysis revealed that normal liver tissue was more likely if high RFU values were recorded with either wavelength and fluorophore combination. Based on a random set of observations, a threshold probability value for distinguishing normal liver from CRLM tissue has been established for the measured CLE data. Subsequent validation on the remaining observations revealed a very good and excellent diagnostic accuracy of 83.9 and 97.8% for the fluorescein/488 nm and ICG/660 nm, combination, respectively. Based on the diagnostic results in the validation datasets, CLE imaging of ICG at 660 nm may be better suited for the use of fluorescence intensity to discriminate between normal and CRLM tissue. The superior performance when using CLE imaging of ICG at 660 nm for this purpose probably reflects the more homogenous nature of CRLM tissue visualised with this CLE setting. As described above, an abundance of high intensity vascular structures seen with CLE imaging of fluorescein at 488 nm causes a heterogeneous imaging pattern in areas of CRLM. Whether a combination of both fluorophore and wavelength combinations can enhance diagnostic accuracy, could not be shown in this study due to the inability of applying simultaneous dual waveband CLE imaging to exactly the same field of view. A new version of Cellvizio™ capable of simultaneous dual waveband imaging is now available but we did not have access to this system for application in this study.

Because probability values used for ROC analysis are based on a combination of a fixed (RFU) and a random effect (animal ID), it cannot be extrapolated into a specific RFU value for future studies. Before this can be considered, it is crucial to standardise RFU value calibration which could potentially result in reproducible and absolute fluorescence units that could be globally applied across different research groups.

Further limitations that have to be taken into account regarding the presented findings pertain to the animal model and the technical characteristics of confocal laser microscopy. The animal model of CRLM that was studied uses a human cancer cell line and a portal venous route of establishing liver metastasis which is the most common route of GI malignancy disseminating to the liver in humans 51. Despite these similarities, research on murine models of malignancy have well‐described limitations when it comes to applying results to a clinical setting 52. A further issue is that experiments have focused on a single cell line of colorectal neoplasia. This cell line was chosen specifically because it exhibits a moderate to well differentiated tumour histology that can display colonic glandular architecture 53 and, therefore, was felt to be more visually distinct on CLE imaging. It is, however, only representing a small spectrum of the histological characteristics that CRLM may exhibit on virtual histology and, therefore, further validation on different colorectal cancer cell lines may be necessary. No formal liver resection was carried out and, therefore, it is difficult to predict if cutting into CRLM tissue would lead to alteration of its fluorescence properties.

A clear trend for lower fluorescence intensity in CRLM compared to healthy liver tissue was especially prominent on CLE imaging of ICG fluorescence, probably, because it has a strong affinity for hepatic tissue. Colorectal cancer liver metastasis is a common indication for surgical resection of liver malignancy in the western hemisphere and is generally regarded as the only curative treatment option for this disease 54, 55. Some of our results may become relevant for this patient population in the future, but it is unlikely that these findings can be directly transferred to primary liver malignancy (e.g. hepatocellular carcinoma), because fluorophore behaviour probably depends on the organ of origin of the cancer. For example, groups examining patients undergoing liver resection for either CRLM or hepatocellular carcinoma, found decreased or increased ICG related fluorescence within cancerous tissue, respectively 14, 56.

Further restrictions that have to be accounted for are related to the technology behind pCLE imaging. Optical imaging modalities including CLE can visualise details down to a sub‐cellular level but at the cost of a limited imaging depth. The maximal imaging depth of the Cellvizio™ probes used by our group was 0–70 μm depending on probe type, which means that only liver cell architecture that is either superficial or adjacent to the resection margin could be assessed. It has been shown that removal of tumour within an area of <1 mm conveys a patient survival benefit in the resection of CRLM 57. Therefore, a potential use of CLE would be to confirm that a resection margin is clear of cancer by probing the cut surface. Because of CLE's limited depth penetration, it should not be considered as a potential substitute for intraoperative ultrasound imaging but more as a complementary modality that expands the borders of intraoperative imaging into the microscopic domain. The Cellvizio™ probes used in this study had a maximal field of view of 600 μm^2^ which may limit its applicability in the clinical examination of liver resection margins which are usually in the cm^2^‐range. It has previously been shown, however, that clinically relevant pCLE imaging with a field of view of approximately 2 mm^2^ 17 is possible. Robotic control of pCLE during laparoscopic liver surgery has been utilised to create even larger fields of view 58. If clinical translation for intraoperative use of pCLE is considered, it would be crucial to encourage the development of probes that offer a field of view of ≥ 1mm^2^, because this would greatly improve the integration of pCLE imaging into the surgical workflow.

Currently, Cellvizio™ probes are only marketed for endoscopic applications and are not certified for intraoperative use. Some of the probes, however, can be fully sterilised and may potentially be used for imaging during laparoscopic liver resection. If a clinical benefit for the intraoperative usage of this system can be defined, sterility issues and clinical re‐certification should not present a major obstacle.

In conclusion, a clinically licensed pCLE system was used to provide a detailed description of discriminatory tissue characteristics in an orthotopic murine model of CRLM. A dual wavelength approach in conjunction with two fluorophores was found to be of benefit because CLE imaging of fluorescein at 488 nm enabled better visualisation of metastatic tissue whereas quantification of ICG mediated fluorescence intensity demonstrated a better potential to objectively discriminate between normal liver and CRLM tissue at a cellular level. Evaluation of simultaneous (i.e. nonsequential) dual waveband imaging and standardisation of fluorescence values is the next crucial step to advance pCLE imaging of liver malignancy.
